# In situ ambient vibration modal analysis of saguaro cacti (*Carnegiea gigantea*)

**DOI:** 10.1002/ajb2.70116

**Published:** 2025-10-21

**Authors:** Jeffrey R. Moore

**Affiliations:** ^1^ Department of Geology & Geophysics University of Utah Salt Lake City UT USA

**Keywords:** ambient vibration, biomechanical characterization, Cactaceae, modal analysis, nondestructive testing, numerical modeling, resonance frequency monitoring, saguaro cactus

## Abstract

**Premise:**

The structural and dynamic properties of columnar cacti are key inputs for stability analyses; however, no previous studies have been able to resolve these properties from full‐scale tests in situ.

**Methods:**

I present an approach using non‐destructive ambient vibration data to measure the resonance properties (modal frequencies and mode shapes) of single‐stem saguaro cacti and resolve key biomechanical properties. I tested the approach on 11 spears in the Tucson, Arizona region, United States.

**Results:**

Saguaro fundamental frequencies ranged between 0.55 and 3.7 Hz with damping ratios of 1.5–2.1%. Additional higher‐order modes were identified below 10 Hz. Fundamental frequencies scaled linearly with the ratio of stem diameter to height‐squared, but deviated from analytical theory due to an observed increase in Young's modulus for taller plants. Calculated ratios between second‐ and first‐order bending frequencies also deviated from beam theory, indicating that stiffness decreases vertically for a given stem, especially for taller spears. These deviations both likely arise from the morphology of internal wooden ribs, which provide the main flexural rigidity. Numerical modeling at one site confirmed the cantilever approximation and height‐dependent stiffness, revealing an empirically derived Young's modulus that decreased exponentially from 10^7^ Pa at the top of the stem to 10^8^ Pa at its base. Twelve days of monitoring at another site showed that frequencies drift with diurnal cycles, suggesting softening of the outer tissue as temperatures warm during the day.

**Conclusions:**

This non‐destructive approach for structural assessment provides valuable data for biomechanical characterization and stability and ecological analyses.

The saguaro cactus [*Carnegiea gigantea* (Engelm.) Britton & Rose; Cactaceae] is a keystone species of the Sonoran Desert, and its most well‐known and iconic plant. Growing up to 24 m high, this giant columnar cactus can live for 200 years and weigh up to 4500 kg (Yetman et al., 2020). Initially growing as a single vertical stem (called a spear), arms develop after several decades as the cactus reaches maturity. Saguaros have a unique stem morphology that allows them to change volume over time, storing large quantities of water during periods of rainfall to withstand subsequent drought. Interior wooden “ribs” provide structural rigidity and strength, while the surrounding cortex and inner pith store water (Niklas and Buchman, 1994; Yetman et al., 2020). A thick, pliable skin armored with rigid spines up to 5 cm long protect the plant. Strong winds during monsoon storms, often combined with increased mass of the cactus, are a major cause of saguaro collapse.

Ambient vibration modal analysis is a nondestructive technique to characterize the structural conditions of a dynamic system, and monitoring of modal properties (i.e., resonance frequencies and damping ratios) can be used to decipher changes in structural conditions over time (Farrar and Worden, 2007). The technique has been used extensively in structural and mechanical engineering, and recently in geological engineering for purposes of vibration risk assessment and structural health monitoring (e.g., Moore, 2018; Finnegan et al., 2022). Ambient vibration measurements are easy to generate and are non‐destructive; a seismometer is simply placed on the structure and left undisturbed for several minutes or hours. Longer‐term monitoring data can extend for days to years. Seismic data are then analyzed for their spectral properties to identify resonance frequencies and damping ratios, and monitor changes over time.

The Euler‐Bernoulli equation for bending of a cantilever beam offers a simple analytical framework to model the resonance properties of a cantilever‐shaped system, such as rock towers, trees, plant stems, or columnar cacti (Falk et al., 1958; Dowding et al., 1983; Niklas and Moon, 1988; Moore and Maguire, 2004). For a beam with circular cross section, the fundamental frequency of a clamped cantilever (*f*_1_) is (Genta, 2009):

(1) $$f_{1}=\frac{1.875^{2}}{8\pi }\;\cdot \;\frac{d}{h^{2}}\;\cdot \;\sqrt{\frac{E}{\rho }},$$

where *E* is Young's modulus, *ρ* is density, *h* is height, and *d* is diameter. This relationship predicts that, for features with similar material properties, fundamental frequencies scale linearly with a geometrical ratio of *d/h* ^2^; this relationship has been shown to be valid for rock pinnacles (Finnegan et al., 2022) and different species of trees (Jackson et al., 2019). When calibrated against field data sets, Equation 1 can be used to predict resonance frequencies or to assess discrepancies that arise due to variations in material properties among a sample population. Equation 1 can also be cast in terms of extrinsic material properties, stiffness (*K*) and mass (*m*), indicating that frequency is expected to change with mass as *m* ^–1/2^, a relation that may be particularly useful for studying the changing water content of columnar cacti.

Application of ambient vibration techniques to study the dynamic properties and structural composition of columnar cacti in situ is new, and the range and variation of resonance frequencies for saguaro stems has not been previously assessed. However, the equipment needed to make such measurements is well developed, and data processing techniques have been honed through analysis of various subjects across a broad range of disciplines (e.g., Niklas and Moon, 1988; Moore and Maguire, 2004; Martin et al., 2020; Finnegan et al., 2022). Moreover, the composition of a saguaro, like other succulents, is amenable to linking resonance frequency changes to mass changes driven by water storage or loss. Interior wooden ribs provide the main structural rigidity (Vogel, 1995; Molina‐Freaner et al., 1998), and rib properties are unlikely to change substantially on daily or seasonal scales. The amount of water held within the succulent cortex, on the other hand, can vary considerably over time, resulting in a bulk mass change for the cactus. Therefore, changes in resonance frequencies, e.g., before and after a large storm, may be indicative of mass changes accompanying water storage and loss.

Here I report on the vibrational properties of saguaro cacti, combining short‐ and longer‐term measurements to perform modal analysis, assess structural properties, and search for signals related to changes in mass over time. This study was motivated by three hypotheses: (1) Resonance frequencies of saguaro spears are a function of height and diameter and follow the analytical trend for a Euler‐Bernoulli cantilever. (2) Modal analysis of saguaro cacti in situ can be used to quantify material properties useful in studies of wind hydrodynamics and stability. (3) Resonance frequency drifts provide a relative measure of mass change in saguaro cacti and can be monitored to quantify water uptake and respiration. I describe new ambient vibration data generated at 11 saguaro spears in the Tucson, Arizona region in the United States to test these hypotheses. As I demonstrate, hypotheses one and two were generally confirmed, while the third remains ambiguous. Results of this study provide the basis for future structural assessment of saguaro cacti including applications such as modeling wind‐induced vibration and failure and ecological monitoring and may be useful more broadly for nondestructive structural assessment of other columnar cacti and plants.

## MATERIALS AND METHODS

**Figure 1:**
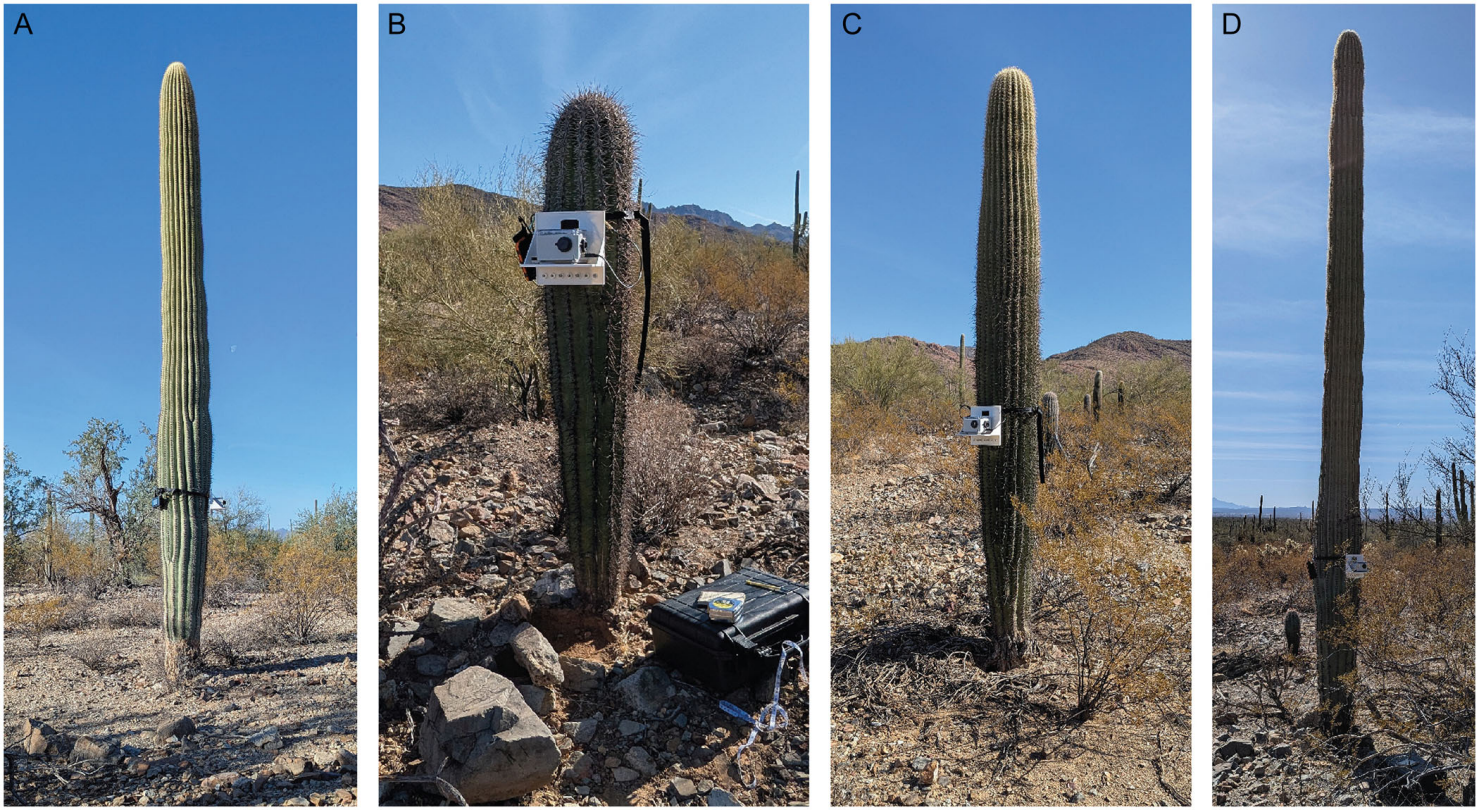
Images of select saguaro cacti: sites (A) O, (B) 2, (C) 3, and (D) 4. See Table 1 for details and Figure 2 for corresponding spectra. The seismometer is attached to each cactus via the white mounting bracket about 1.5 m or less from the ground.

Eleven saguaro cacti were selected for analysis in this study (Table 1). Sites O and 1–9 were located on the west‐southwest‐facing bajada of the Tucson Mountains, west of Tucson, Arizona, at approximately 800 m a.s.l. within Tucson Mountain Park (surveyed with permission). This area was selected for its abundance of saguaros in close proximity, relatively level ground, and similar ground conditions from plant to plant. Site H was located on the south‐facing slope of the foothills of the Catalina Mountains, north of Tucson on private property at a similar elevation. Saguaro cacti studied had heights (*h*) from 0.74 to 7.16 m, with basal stem diameters (*d*) from 0.11 to 0.40 m (Table 1). All were spears (Figure 1). Initial ambient vibration experiments were carried out within 48 h when weather conditions were consistent (average daily temperature = 16°C, no rainfall).

**Table 1:** Geometric and ambient vibration spectral data for the 11 saguaro cacti assessed in this study. Damping ratios (*d*_1_) could only be calculated for study sites with >1 h data. Diam., diameter; Freq., frequency.

Site	Diam. (m)	Height (m)	*d*/*h* ^2^ (m^−1^)	*f*_1_ (Hz)	*f*_2_ (Hz)	*f*_3_ (Hz)	*f*_4_ (Hz)	*d*_1_ (%)	Freq. ratio 1	Freq. ratio 2
O	0.29	5.26	0.010	0.90	0.93	3.6	3.7	1.5	4.0	4.0
1	0.24	3.53	0.019	0.93	1.0	4.9	5.4	–	5.3	5.3
2	0.16	1.65	0.058	1.4	1.5	8.1	8.9	–	5.7	5.8
3	0.24	3.05	0.026	1.2	6.4	–	–	–	5.2	–
4	0.32	6.81	0.007	0.58	2.3	5.1	8.8	–	4.0	–
5	0.11	0.74	0.211	3.7	4.4	–	–	–	–	–
6	0.31	4.14	0.018	1.2	1.3	4.4	5.2	–	3.7	4.1
7	0.22	2.13	0.048	1.7	1.8	7.9	10	–	4.7	5.6
8	0.40	7.16	0.008	0.55	0.56	2.0	2.0	–	3.6	3.6
9	0.29	3.48	0.024	1.1	1.3	5.3	6.0	–	4.6	4.7
H	0.21	2.10	0.047	1.5	1.8	7.0	9.7	2.1	4.5	5.3

Each saguaro was temporarily instrumented with a Raspberry Shake 3D seismometer. The Raspberry Shake 3D is a low‐cost, lightweight three‐component seismometer, that has been extensively benchmarked against other instruments (e.g., Arosio et al., 2023). It records three mutually orthogonal components of motion (two horizontal and one vertical) using 4.5‐Hz geophones with range electronically extended down to at least 0.5 Hz, at a sampling rate of 100 Hz, and internal data storage. I devised a custom mounting bracket consisting of a 3D‐printed platform that could be strapped to a saguaro stem and leveled about 1.5 m above ground level (Figure 1). The mounting height was arbitrary, being as high as feasible without requiring a ladder, and the mounting method ensured that the cactus was not damaged. A small portable battery provided power.

Ambient vibration data were recorded at study sites 1–9 (Table 1) for approximately 15 min each, while sites O and H had longer deployments of >1 h. Seismic data were processed for spectral attributes by first removing the mean and trend from each trace, then removing the instrument response, and finally computing power spectra density (PSD) estimates using overlapping fast Fourier transforms in the band between 0.2 and 40 Hz. Damping ratios (given in % of critical) describe the rate of energy loss from a dynamic system, which can be caused by internal and external mechanisms such as friction and root–ground interactions, and were estimated using the random decrement technique (Cole, 1973; Guéguen et al., 2016; Geimer et al., 2022). Damping could only be determined from ambient vibration data for sites with ~1 h of data.

Measurements of saguaro geometry, including height and basal circumference, were made in the field using a tape measure. Determining stem circumference by this method is made difficult by the presence of thorns, but while thorn density varied from stem to stem, most had fewer thorns near the base. Stem height was easily measured for short cacti, but the height of tall stems may have an error of several centimeters. Repeated measurements from several sites indicate stem diameter is accurate to within ±1 cm, while stem height measurements have maximum uncertainty of ±5 cm. One study site, site O, was additionally subject to 3D photogrammetric reconstruction using a series of ground‐based images taken while orbiting the cactus with the aid of a long pole. I used Context Capture from Bentley Systems (Exton, PA, USA) for photogrammetric model generation, and Autodesk's (San Francisco, CA, USA) Meshmixer for model refinement, including trimming, smoothing and transitioning the surface mesh to a solid object. Model scaling was verified against field measurements.

Numerical modal analysis was performed for site O using the finite‐element software COMSOL Multiphysics. I imported the 3D geometry generated from photogrammetry, then applied fixed boundary conditions to the ground around the base of cactus. Eigenfrequency analysis is solved in the frequency domain; no time history input or excitation is applied. Required material properties are Young's modulus (*E*) and density (*ρ*). I assumed *ρ* was constant at 800 kg/m^3^ (Niklas and Buchman, 1994) and let Young's modulus—which is an unknown bulk value representing the combined elements of the plant—vary using the approach of Moore et al. (2018) and Finnegan et al. (2022). Different assumptions were tested for *E*, from a constant isotropic value to values that varied with stem height. Sensitivity analyses were also performed implementing different density values and simplified geometric shapes.

Saguaro site H (Table 1) was selected for an exploratory monitoring experiment in which continuous ambient vibration data were recorded for 12 days. The Raspberry Shake was installed as previously described, with horizontal components arbitrarily oriented (EH1 azimuth = 340°). Resonance frequency tracking was performed following methods of Geimer et al. (2022). Air temperature was recorded on site using an Onset HOBO U23 system with radiation shield, and rainfall data were from a Pima County gaging station 1 km away (no rain fell during the experiment). After 5 days of dry conditions, an artificial watering experiment was performed where ~75 L of water were sprinkled over an area of 3.2 m^2^ around the base of the stem over 20 min. The water volume and area resulted in an average water application of ~2 cm, simulating rainfall during a monsoon storm.

## RESULTS

**Figure 2:**
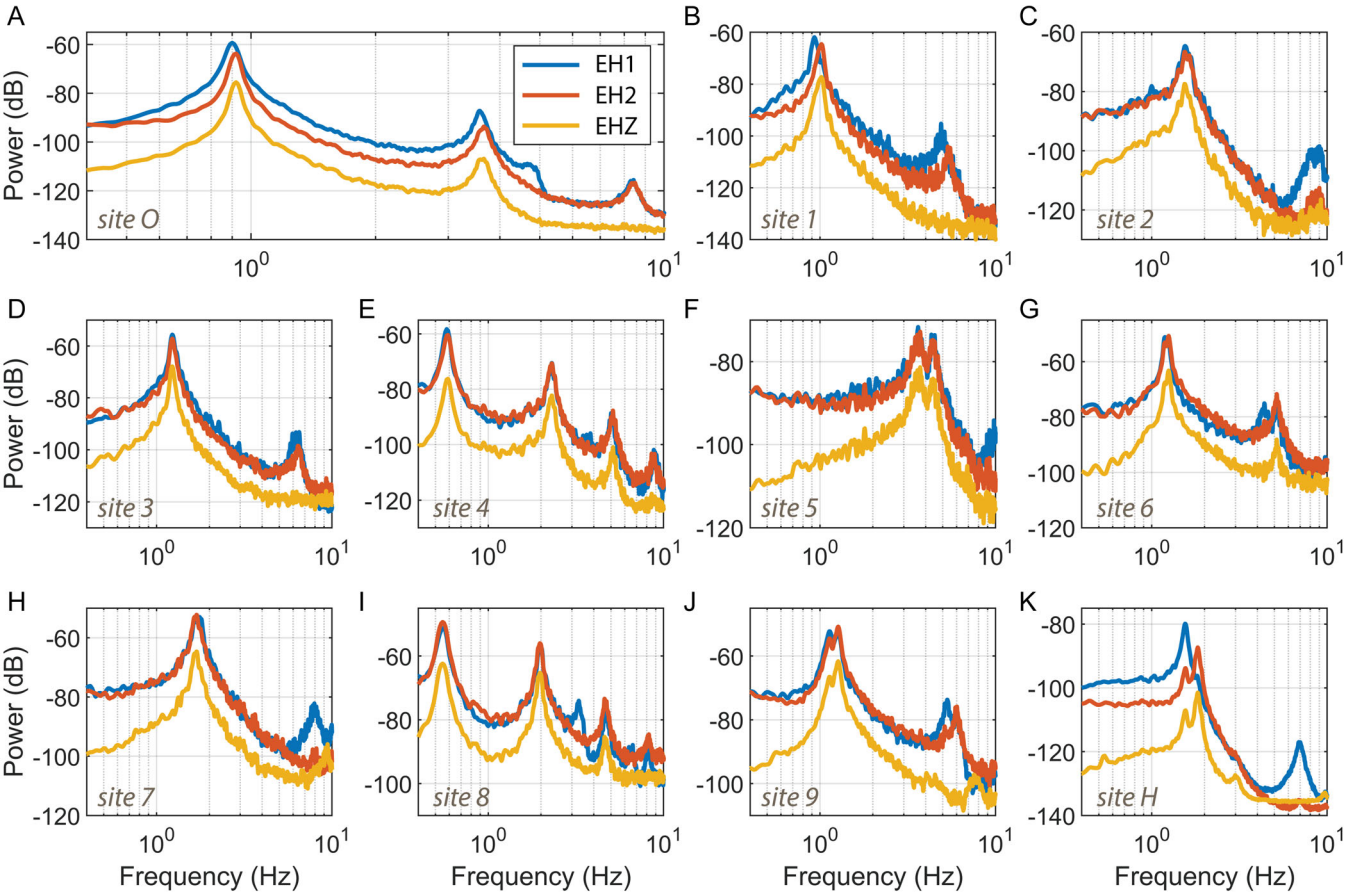
Power spectral density plots for (A–K) the 11 saguaro cacti assessed (see also Table 1). Power is shown relative to 1 m^2^ s^–2^ Hz^–1^. EH1 and EH2 are two orthogonal components of horizontal motion, which have arbitrary orientation unless specified (site O: EH1 azimuth = 50°). EHZ is vertical. Spectra from sites O and H were based on ~1 h of data; sites 1–9 used just 15 mins.

**Figure 3:**
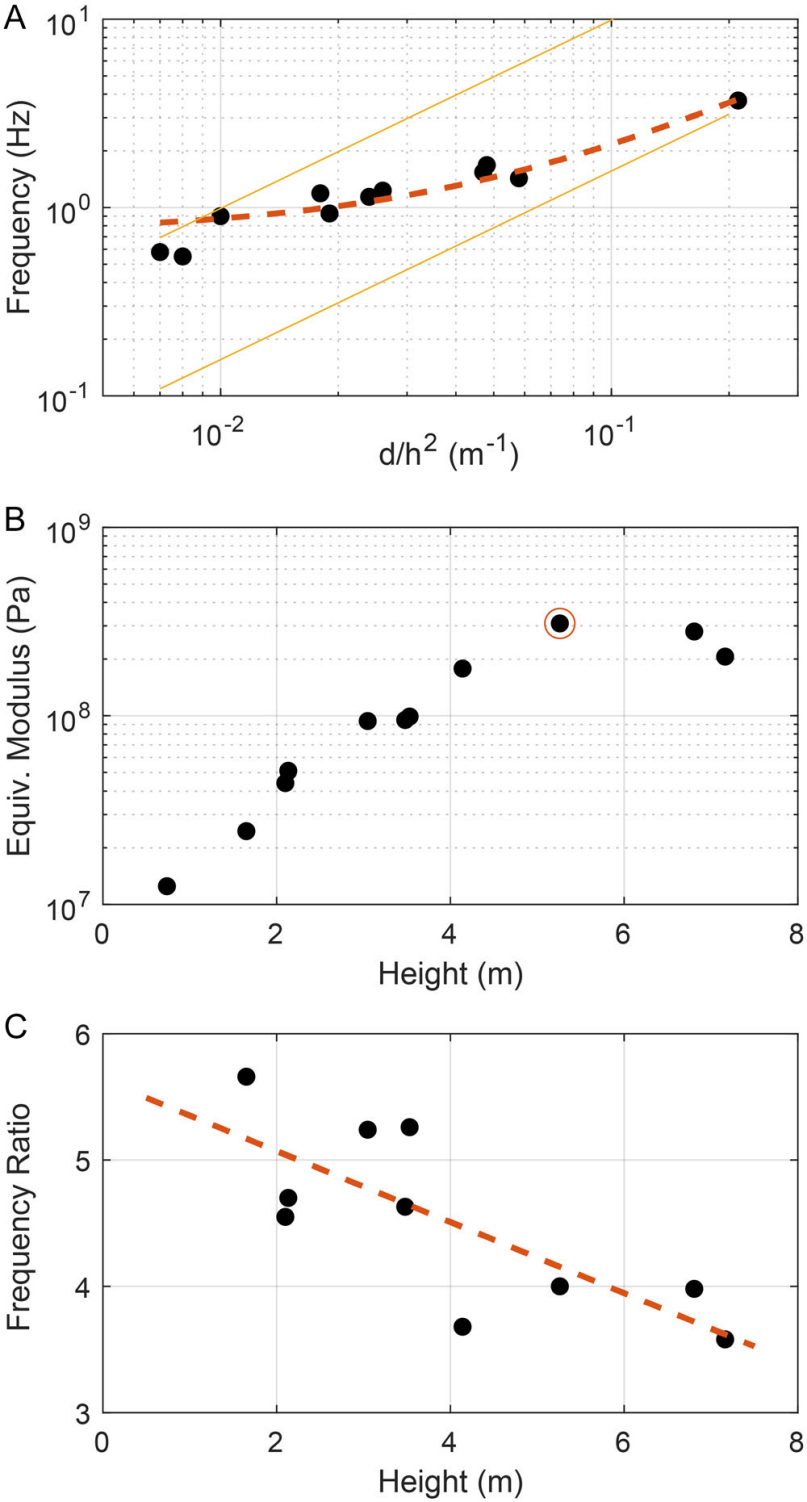
(A) Fundamental frequency for the 11 study sites versus ratio of basal diameter to height‐squared (Table 1). Best‐fit linear trend shown (dashed line, see Equation 2; *R* ^2^ = 0.96), along with upper (*E* = 4 × 10^8 ^Pa) and lower (*E* = 1 × 10^7 ^Pa) bounds determined from Equation 1 with constant density. (B) Equivalent Young's modulus (calculated from Equation 1) compared to stem height. Site O highlighted (cf. Figure 4). (C) First frequency ratio plotted against cactus height, showing inverse linear trend (*R* ^2^ = 0.58).

Ambient vibration spectral analysis of 11 saguaro cacti revealed fundamental resonance frequencies (*f*_1_) between 0.55 and 3.7 Hz (Table 1, Figure 2). All results showed largest motions on the horizontal components, indicating dominantly horizontal deflection, with minor contributions of vertical motion. Moreover, data often showed two closely‐spaced first modes, such as at saguaro site O with *f*_1_ = 0.90 Hz and *f*_2_ = 0.93 Hz. This close similarity is expected for objects with near radial symmetry and is caused by slight deviations in geometry in different orientations (Martin et al., 2020). Comparison of geometrical and resonance frequency data (Figure 3A) revealed that saguaro fundamental frequencies scale linearly with the ratio of *d*/*h* ^2^, but deviate from the analytical prediction in Equation 1 with the inclusion of a *y*‐intercept. The empirically derived best fit to field data was:

(2) $$f_{1}=14.38\frac{d}{h^{2}}+0.73.$$

Some scatter from the trend was found among sites but the overall agreement was good (*R* ^2^ = 0.96). Damping ratios determined for the fundamental mode of sites O and H were 1.5% and 2.1%, respectively.

The deviation of saguaro fundamental frequencies from simplified beam theory (Equation 1) suggests non‐uniform material properties among the sample population. To explore this idea, I calculated the “equivalent” Young's modulus (*E*_eq_) for each site, using Equation 1 and the measured fundamental frequencies and geometric ratios. I assumed a constant bulk density of 800 kg/m^3^ (Niklas and Buchman, 1994), a value which is not precisely known but is unlikely to vary by more than ±10%. Results showed a clear trend of increasing equivalent Young's modulus with spear height (Figure 3B), indicating taller spears are generally stiffer than shorter spears. Interestingly, the limited data also showed an apparent trend where the tallest spears do not necessarily have the greatest equivalent Young's modulus; instead the peak value was found at site O (Figure 3B, Table 1).

Several higher frequency modes up to 10 Hz were measured for all spears, and in many cases, four modes were evident (Figure 2). These have lower power than the fundamental mode, indicating lower levels of excitation. A useful metric is the ratio between second‐ and first‐order cantilever bending frequencies, which for a beam of uniform geometry and material properties is theoretically 6.27 (Genta, 2009). A second‐order mode is one that has a single node point, or point of zero displacement, located in the upper part of the beam. Table 1 shows the first frequency ratios, calculated as *f*_3_/*f*_1_ when two closely spaced first modes were observed, otherwise *f*_2_/*f*_1_. The second frequency ratio is *f*_4_/*f*_2_ when two first‐order modes were observed. Frequency ratio values were ~4–5, notably less than that predicted for a uniform cantilever, suggesting non‐uniform material properties. Comparison also shows that the first frequency ratio varied systematically with spear height (Figure 3B), decreasing for taller cacti.

**Figure 4:**
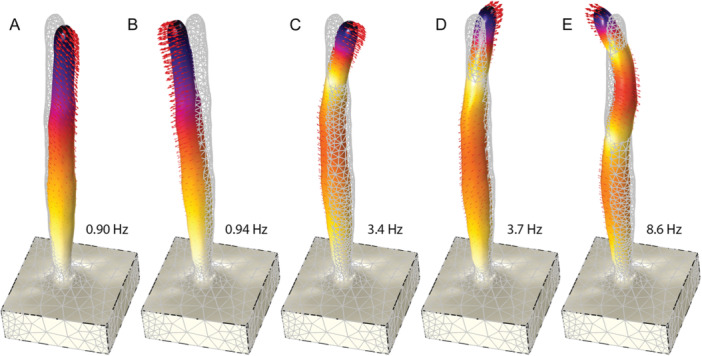
Numerical eigenfrequency analysis showing the first five predicted resonance modes for site O (compare to measurements in Table 1). Model assumes constant density of 800 kg/m^3^ and variable Young's modulus that decreases exponentially from 4 × 10^8 ^Pa at the base to 1 × 10^7 ^Pa at the top. Colors and arrows show exaggerated modal displacement; gray wireframe is the reference form. (A, B) First‐order bending modes, (C, D) second‐order, (E) third‐order mode shape. Modal animations are presented in Appendix S1.

Numerical modal analysis predicted the first several modes of vibration for saguaro site O (Figure 4; Appendix S1). The accurate geometry implemented (albeit smoothed) meant that material properties were the main unknown. A first test implementing uniform material properties matched the first two resonance frequencies of the spear, but overestimated the third and fourth, with frequency ratios around 7 (i.e., greater than a uniform cantilever). Several empirical tests implemented a height‐dependent Young's modulus, rooted in the observation that saguaro ribs thin in diameter vertically. I found that an exponential decrease in Young's modulus from the base to top of the spear could reproduce measured resonance frequencies (Figure 4), in this case, closely matching the five measured modes (compare Table 1 and Figure 4; note that *f*_5_ for the spear was measured at 8.6 Hz, which is not shown in the table). Modelled modal vectors also matched well for *f*_1_ where they could be determined from field data (315° azimuth predicted and 311° measured). Implemented *E* decreased from 4 × 10^8 ^Pa at the base of the spear to 1 × 10^7 ^Pa at the top.

**Figure 5:**
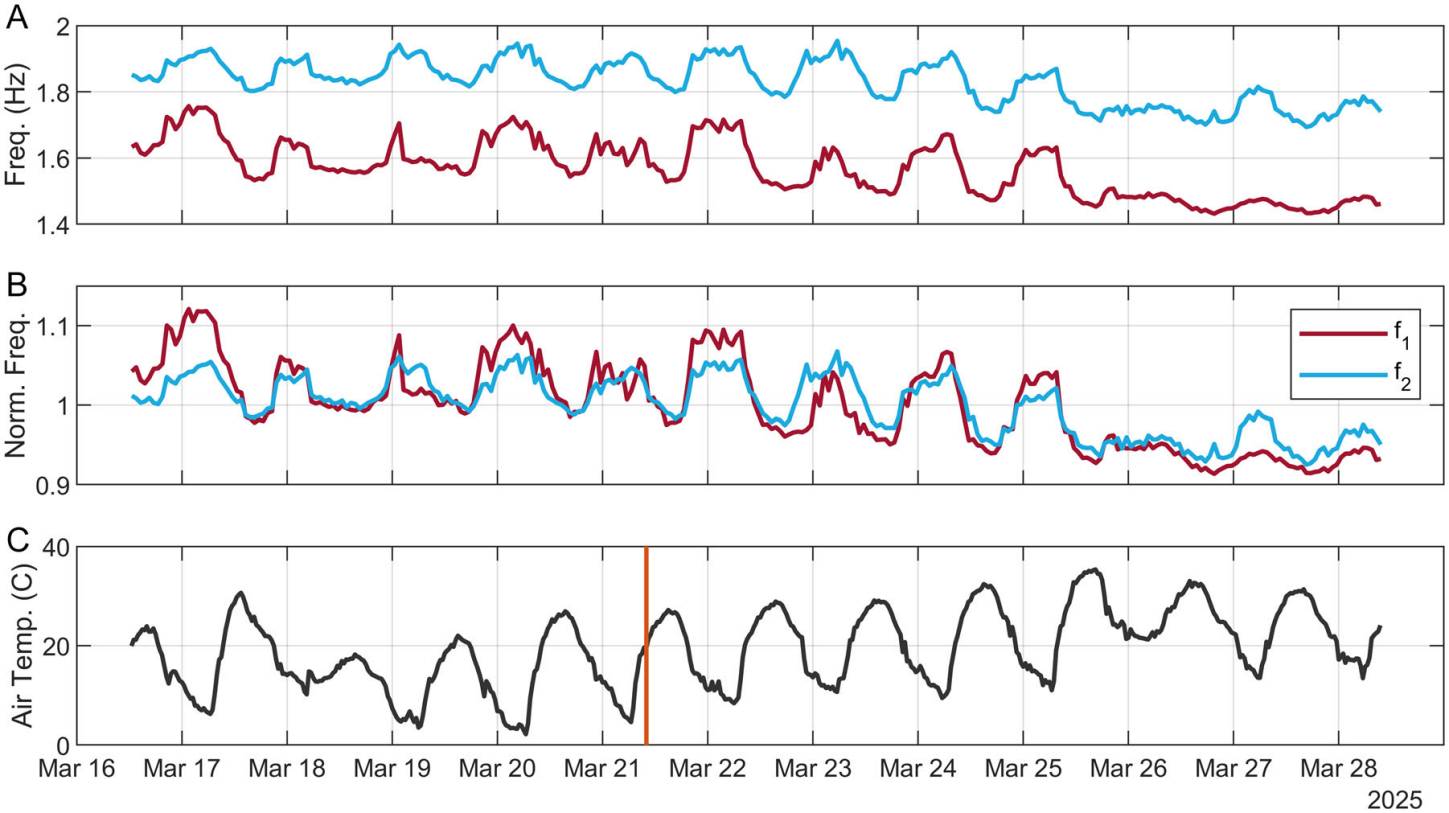
Resonance frequency drifts at saguaro site H over 12 days of monitoring: (A) raw and (B) normalized by mean. (C) Local air temperature; orange bar indicates watering time. Times are MST.

Monitoring at saguaro site H started on 16 March 2025 12:00 hours local time (MST) and ended on 28 March 2025 at 09:00 hours. Ambient conditions prevailed for the first 5 days (Figure 5), and then 75 L of water were sprinkled at the base of the cactus on 21 March 10:00 hours MST. There was no natural rainfall. Resonance frequency monitoring showed a clear diurnal drift in both the first and second frequencies (Figure 5), with frequencies decreasing during the day and increasing at night. This pattern closely mimicked the inverse of temperature; i.e., resonance frequencies decreased as temperatures warmed during the day, and vice versa, with no observable phase lag. Daily frequency changes were in the range of 5–10%, generally smaller for *f*_2_ and greater for *f*_1_, which may be related to varying sun exposure on different aspects of the stem (Dzubay et al., 2022). Similarly, some days showed little frequency change, which may be a function of cloud cover reducing insolation. Application of water on the morning of 21 March had no clear effect on resonance frequency drifts; an increase in mass would manifest as a decrease in frequency, which was not observed (Figure 5).

## DISCUSSION

Results of this study demonstrate that the Raspberry Shake 3D is suitable for measuring the dynamic properties of saguaro cacti. The range of resonance frequencies determined, generally between 0.5 and 10 Hz, is within the range of accuracy and sensitivity for the instrument (Arosio et al., 2023), and the relatively high levels of ambient excitation (velocities typically ~mm/s) for the pliable stems mean the measurements are not pushing the Raspberry Shake noise floor (Figure 2). Moreover, its low mass (~0.6 kg) helps ensure mounting did not cause damage to the fragile saguaro skin. However, mounting the seismometer to a vertical stem required a custom bracket (Figure 1), and my data suggest that this bracket with the weight of the instrument has its own vibrational modes. Specifically, I observed spectral peaks in the range of 14–15 Hz in measurements from all sites, which I attribute to the mounted device. These modes provided an upper limit on the assessed resonance frequencies for the cacti, which could be improved with future bracket designs.

Many natural objects (e.g., rock towers, stalagmites, plant stems, trees) have resonance frequencies in the range of 0.5–10 Hz (Niklas and Moon, 1988; Moore and Maguire, 2004; Martin et al., 2020; Finnegan et al., 2022), and to the first order, objects shaped like cantilevers conform to the relationship *f*_1_ ∝ *d*/*h* ^2^. My saguaro data generally agree with this expectation, but the empirical linear fit (Equation 2) deviates from beam theory with the inclusion of a *y*‐intercept. This finding suggests that material properties vary among the test population, and indeed, my analysis (Figure 3B) showed that the equivalent Young's modulus (calculated from Equation 1) is greater for taller spears. This effect is likely rooted in the changing diameter of internal wooden ribs, which provide the main structural rigidity for saguaro stems; taller cacti have thicker ribs especially near the base of the stem where bending stresses are largest. The calculated equivalent Young's modulus can be compared to my numerically derived value for saguaro site O: *E*_eq_ = 3 × 10^8 ^Pa, which is similar to the model derived value of 4 × 10^8 ^Pa at the base of the stem. The equivalent modulus thus best represents the Young's modulus near the stem base, which is expected since flexural strain is greatest at the base of a bending cantilever.

Comparisons between second‐ and first‐order resonance frequencies show deviations from the expected theoretical value for a uniform cantilever of 6.27, with most measured values for saguaro spears in the range of 4–5 (Table 1). These deviations can be caused by numerous factors, including density or stiffness variations and geometry (Niklas and Moon, 1988). To investigate controlling parameters, I performed a series of numerical simulations. Simple first tests using conjoined conical shapes indicated that a bulging midsection (as often seen in saguaro stems) caused the frequency ratio to increase, rather than decrease, and that only a uniform decrease in diameter with height (i.e., a linearly tapered beam) resulted in a decreasing frequency ratio, a form not observed in natural spears. In subsequent tests using the model for site O, with its accurate stem geometry (Figure 4), I varied density as a function of stem height. I found that implementing a height‐dependent density decrease in the range of 10% had no effect on resonance frequency ratios. Only simulations that implemented an exponentially decreasing Young's modulus with stem height generated a notable reduction in frequency ratios that matched field data. This finding is similar to that of Niklas et al. (1999) based on destructive testing of xylem strands from a similar large columnar cactus, *Pachycereus pringlei*.

The result that a decreasing Young's modulus with stem height is required to match resonance frequency measurements (Figure 4), and specifically data from site O, suggests that saguaro stems soften vertically. Additionally, data shown in Figure 3B indicate that taller spears have preferentially softer tops (seen as lower frequency ratios). This observation can be reconciled considering the morphology of saguaro ribs, which provide the main structural rigidity (Molina‐Freaner et al., 1998): Ribs decrease in diameter from the base to the top of the stem. Simplified conceptual evaluation of the stiffness of an individual rib as *K* = *EI*, where *I* is the moment of inertia for a circular cross section, and *E* is the Young's modulus for wood, and assuming rib diameter decreases linearly with stem height *h*, suggests that rib stiffness decreases as *h* ^4^. This decrease is not far from my empirically determined exponential decrease in Young's modulus modelled for site O (Figure 4). It is important to note that the modulus implemented in my model represents the bulk effect of the ribs, cortex, pith, and skin combined over the full height of the plant. In addition, although the ribs likely are the dominant contributor to stiffness, other tissues likely also contribute. For comparison with my model‐determined Young's modulus (range 10^7^–10^8 ^Pa), Niklas and Buchman (1994) reported that the Young's modulus of individual saguaro rib wood segments is in the range of 10^10 ^Pa. This difference is expected because the ribs occupy only a small fraction of the total cross‐sectional stem area.

Hydrodynamic experiments and simulations are increasingly utilized to analyze the conditions and controls on wind‐induced vibration that may lead to saguaro collapse (e.g., Talley and Mungal, 2002; El‐Makdah and Oweis, 2013; Lin et al., 2020). Such methods rely on accurate description of saguaro material and vibrational properties; however, prior to now, no studies were available that provided suitable input parameters for these simulations based on full‐scale, in situ field tests. The results I report thus represent valuable input parameters for hydrodynamic simulations, testing saguaro response to different wind conditions, and analyzing conditions for wind‐induced toppling failure. Interesting results from past simulations suggest the star‐shaped stem cross section and spines play an important role in improving wake behavior as wind flows around a saguaro, reducing drag forces and limiting lateral forces caused by vortex shedding (Talley and Mungal, 2002; El‐Makdah and Oweis, 2013). The modal analysis, material properties, and structural characterization results reported here can contribute to more accurate future simulations of saguaro wind response. However, important differences might arise in material and dynamic properties for large‐strain swaying during strong winds as compared to the ambient vibration results presented here (Miller, 2005).

While my study demonstrated that key aspects of biomechanical characterization of saguaro stems can be obtained from ambient vibration data, this approach does have limitations. Foremost, the cantilever approximation and beam theory analysis can only be applied for single‐stem spears, limiting the sample population that can be tested. In addition, substrate conditions can vary spatially for different geologic materials or temporally, for example, during rainstorms, affecting root–ground interactions. These factors could lead to differences in resonance properties for similarly shaped stems, and in the extreme, ground softening might invalidate the clamped cantilever assumption, leading to rocking motion. Moreover, all data obtained in this study are small‐amplitude ambient motions and thus represent small‐strain conditions; however, the stem response under large‐strain conditions (e.g., high winds) may differ (Miller, 2005). Plant tissue can have strain‐rate‐dependent viscoelastic properties (Vincent, 1990); therefore, deviations may occur between the measured resonance properties reported here and during high‐amplitude excitation events. In addition, damping ratios reported for two sites in this study under small‐strain ambient conditions are low (cf. Moore and Maguire, 2004; Miller, 2005), and could increase at higher strain rates (Guéguen et al., 2016), facilitating energy dissipation during storms. Root–ground interactions may similarly affect resonance frequencies and damping ratios as ground moisture conditions change over time (frequencies are expected to decrease for water‐softened soil). Further measurements and new monitoring experiments are needed to explore these factors.

Resonance frequency monitoring over 12 days at saguaro site H revealed clear diurnal drifts for *f*_1_ and *f*_2_, with daily changes in the range of 5–10% (Figure 5). Interestingly, frequencies rose overnight as temperatures cooled and decreased during the day as temperatures warmed. This pattern is opposite to that reported in most past studies of rock features, such as rock arches, where frequencies increase during warming as the rock undergoes thermal expansion (Geimer et al., 2022). Equation 1 provides the basis to interpret resonance frequency drifts: Either mass increases or the stem becomes softer (lower elastic modulus) during the day, resulting in decreasing frequencies, with reversible cycling each night. The mass change needed to create a 5–10% frequency decrease would be in the range of 10–20%, which is unlikely for a diurnal change. Therefore, resonance frequency drifts may instead be driven by diurnal stiffness cycles, where the stem becomes stiffer overnight with cooler temperatures and softer during the day with warmer temperatures. Similar as mass, the observed frequency change suggests a 10–20% daily variation in stem stiffness. Since such large diurnal stiffness changes are unlikely for wooden ribs and as I observed no phase lag between temperature and frequency, this change most likely arises from the succulent cortex near the stem surface, suggesting that the pliability of this material is thermally modulated.

The watering experiment produced no clear change in resonance frequencies beyond the nominal temperature‐associated drifts. The amount of water I applied was an estimated 75% of the saguaro mass, part of the reason a smaller spear was selected for testing. If the cactus were able to take up just 25% of this amount, mass would increase by ~20% and frequency would be expected to decrease by ~10%. While I observe decreases in *f*_1_ and *f*_2_ of ~7–9% over the 12 days of monitoring, these are gradual and delayed from the watering, and it is unclear if this change is outside the expected effect from gradual warming (Figure 5). Thus, the hypothesis that resonance frequency drifts can be used to detect and quantify mass changes from water uptake was not confirmed. A plausible reason why no clear frequency decrease was generated by the experiment was that the particular saguaro at the time of year tested was not primed for or in need of a rapid uptake of water. My measurements were conducted in March, at the end of the rainier winter season and well before the hottest summer months or subsequent monsoon (July–September), and ~10 mm rain fell on the days prior (11 and 12 March). Thus, future experiments should be conducted during hot summer months and/or the monsoon season. Another possible reason is masking of the signal by competing effects, i.e., an increase in mass is accompanied by an increase in stiffness (Equation 1), nullifying the net change in frequency (cf. Falk et al., 1958). Additional experiments are recommended to explore this hypothesis.

The methodology I present here is useful not only for ongoing studies of saguaro cacti, but also broadly across different species of columnar plants (e.g., Molina‐Freaner, 1998). This study could be extended with future measurements of cacti with branching arms or groups of stems, moving beyond the single cantilever simplification to explore the full range of resonance frequencies and mode shapes associated with complex morphologies. Other areas of future work could include a larger data set with stems in different substrates or longer monitoring that included summer and monsoon periods. One key advantage of this field approach is that it is nondestructive and non‐invasive, causing no permanent damage to the plant, in contrast to other past approaches for biomechanical characterization. Additionally, ambient vibration methods used here are similar to those already in practice to describe the sway frequency and damping ratios of trees (e.g., Moore and Maguire, 2004), which share similarities to the cantilever approximation but differ in their weighted and branching upper canopy. Common factors, however, include aspects such as root–ground interactions that can affect damping ratios and are likely to vary seasonally. With the advent of increasingly inexpensive and lightweight, high‐performance seismometers, and well‐established methods of spectral analysis, the ability to generate ambient resonance data in situ is increasingly feasible, and further exploration of the utility of these measurements may open new possibilities in plant ecology research.

## CONCLUSIONS

I used ambient vibration data to perform modal analysis of 11 in situ saguaro spears in the Tucson, AZ region. Results revealed fundamental frequencies between 0.55–3.7 Hz and damping ratios in the range of 1–2%. I hypothesized that Euler‐Bernoulli beam theory would provide a suitable analytical framework for assessing the dynamic properties of saguaro spears, which was generally confirmed but with interesting deviations from theory relating to variable Young's modulus among the sample population (taller spears are stiffer). Moreover, I found that frequency ratios comparing second‐ to first‐order bending modes were lower for saguaro spears than expected for a uniform cantilever, due to varying material properties for a single stem (taller spears have softer tops). Both effects are likely rooted in the morphology of interior wooden ribs, which provide the main flexural rigidity for saguaro stems. I then conducted 3D numerical eigenfrequency analysis at one site, using photogrammetry to accurately reconstruct the stem geometry. Numerical results confirmed the basic cantilever hypothesis (i.e., mode shapes), and the best match to field data was achieved when I implemented an exponentially decreasing Young's modulus with stem height (values ranging from 4 × 10^8 ^Pa at the base to 1 × 10^7 ^Pa at the top). I finally hypothesized that resonance frequency monitoring might provide a relative measure of mass change in saguaro spears and tested this hypothesis in a 12‐day experiment at one site where a large amount of water was introduced at a known time. Results did not show the expected decrease in resonance frequency associated with an increase in mass, as predicted by beam theory. Rather, the data indicated relatively large diurnal frequency drifts, which I propose are caused by stem tissue softening during the day as temperatures warm and stiffening with nighttime cooling. This study provides a new, non‐destructive means of assessing the structural and dynamic properties of columnar cacti, with results that may be particularly useful in hydrodynamic stability analyses and ecological monitoring.

## AUTHOR CONTRIBUTIONS

J.M.: conceptualization, funding acquisition, formal analysis, investigation, methodology, visualization, writing original draft, review, and editing.

## Supporting information


**Appendix S1.** Modal animations (cf. Figure 4) for the first six resonance modes of saguaro site O. Model assumes constant density of 800 kg/m^3^ and variable Young's modulus that decreases exponentially from 4 × 10^8^ Pa at the base to 1 × 10^7^ Pa at the top. Colors and arrows show exaggerated modal displacement; gray wireframe is the reference form.

## Data Availability

Data generated in this study can be accessed from the University of Utah HIVE repository: https://doi.org/10.7278/S5d-kx51-x9dg.
